# Global region-specific influenza early warning by establishing an epidemic intensity threshold system: a modeling study

**DOI:** 10.3389/fpubh.2026.1885724

**Published:** 2026-08-04

**Authors:** Zhou Guan, Jinsong Gao, Yuetong Li, Jinna Wang, Mingyu Luo, Qinmei Liu, Tianqi Li, Zhenyu Gong, Jimin Sun

**Affiliations:** 1 Key Laboratory of Vaccine, Prevention and Control of Infectious Disease of Zhejiang Province, Zhejiang Provincial Center for Disease Control and Prevention, Hangzhou, China; 2 School of Public Health, Hangzhou Medical College, Hangzhou, China; 3 Department of Epidemiology, School of Public Health, Fudan University, Shanghai, China

**Keywords:** early warning, epidemic intensity thresholds, influenza, moving epidemic method, peak timing simulation

## Abstract

**Background:**

Influenza exhibits marked geographical heterogeneity in epidemic patterns, yet there is no globally standardized approach for determining epidemic thresholds. This study aimed to establish a region-specific epidemic intensity threshold system and evaluate its performance in influenza early warning and peak timing simulation.

**Methods:**

Weekly influenza surveillance data from 167 countries (2009–2020) were obtained from the WHO Global Influenza Surveillance and Response System (GISRS). Three indicators were calculated for each of the 18 influenza transmission zones: ILI consultation rate (ILI rate), influenza virus positivity rate (IV positivity rate), and influenza incidence. MEM models were fitted according to each zone’s epidemic pattern to establish zone-specific threshold systems. Early warning and peak timing simulation performance were evaluated across target seasons. Model performance was assessed using leave-one-out cross-validation.

**Results:**

Three epidemic patterns were identified: seasonal single-peak (North America, Europe, and Temperate South America), mixed single- and dual-peak (East Asia), and year-round or irregular (most African and tropical zones). Zone-specific four-level intensity thresholds were derived. The IV positivity rate demonstrated the most robust early warning performance, with alert-to-epidemic-start differences within ±2 weeks in most zones. Exact concordance or ±1 week difference accounted for the majority of observations. Peak timing simulation using influenza incidence achieved errors within ±3 weeks in 11 of 18 evaluable observations, concentrated in Europe and East Asia. Model sensitivity and specificity exceeded 80% in zones with distinct seasonality. The ILI rate performed adequately only in data-rich zones and showed limited utility in tropical and African settings.

**Conclusion:**

The region-specific threshold system achieved early warning within ±2 weeks for the IV positivity rate in most zones, and peak timing errors within ±3 weeks in 11 of 18 evaluable observations, with sensitivity and specificity exceeding 80% in zones with distinct seasonality. The IV positivity rate is the most reliable indicator for global applications, while ILI-based indicators are best suited to zones with robust surveillance infrastructure and distinct seasonality. These findings support tailoring influenza early warning strategies to local epidemic characteristics.

## Introduction

Influenza is an acute respiratory infectious disease caused by influenza viruses, characterized by rapid transmission and widespread susceptibility, leading to seasonal epidemics and occasional pandemics worldwide (1, 2). Annually, an estimated 5–10% of adults and 20–30% of children are infected globally, resulting in approximately 291,000 to 645,000 excess deaths (3). Although global influenza surveillance networks, notably the Global Influenza Surveillance and Response System (GISRS) coordinated by the WHO, have achieved substantial progress (4), critical challenges remain in translating surveillance data into effective early warning systems and dynamically optimized prevention and control strategies (5, 6).

A central challenge lies in the marked geographical heterogeneity of influenza epidemic patterns (7, 8). In temperate regions, influenza activity exhibits clear seasonal peaks, typically during winter months (9). In contrast, tropical and subtropical regions display more complex patterns, with epidemic peaks potentially occurring at multiple time points throughout the year (10, 11). This spatiotemporal variability, shaped by climatic, demographic, and socioeconomic factors, complicates the development of standardized prediction and early warning tools applicable across diverse settings (12).

Most existing influenza prediction models rely on a critical common step: comparing modeled epidemic activity against a pre-specified epidemic threshold, with an alert triggered once the threshold is reached or exceeded (13–15). The validity and timeliness of early warning therefore depend fundamentally on the appropriateness of the threshold. However, there is currently no globally standardized approach for determining influenza epidemic thresholds, and methods vary considerably across countries and regions. Some settings have empirically adopted a 10% influenza virus positivity rate as the epidemic threshold (16, 17), an approach that may inadequately capture the dynamic nature of influenza epidemics and fail to account for cross-regional differences in surveillance intensity and capacity.

The Moving Epidemic Method (MEM), recommended by the European Centre for Disease Prevention and Control (ECDC) and the WHO for influenza surveillance and threshold evaluation, offers a data-driven framework for establishing epidemic and intensity thresholds using historical surveillance data (17–19). MEM has been validated and applied in multiple countries, predominantly in temperate and subtropical settings (16, 18, 20–22). However, its application across the full spectrum of global influenza transmission zones, particularly in tropical and African regions with year-round or irregular epidemic patterns, remains largely unexplored (19).

In 2018, the WHO classified countries and territories into 18 influenza transmission zones based on shared patterns of influenza activity. This classification provides a systematic geographical framework for investigating region-specific epidemic dynamics and for developing tailored threshold systems. Building on this framework, the present study aims to: (1) analyze influenza epidemic patterns and periodicity across global transmission zones using comprehensive surveillance data from GISRS; (2) establish region-specific epidemic intensity threshold systems using MEM, adapted to the distinct epidemic patterns of each zone; and (3) evaluate the performance of these thresholds in influenza early warning and epidemic peak timing simulation, thereby informing the optimization of influenza vaccination strategies and broader prevention and control efforts.

## Methods

### Data source

Influenza surveillance data were obtained from two databases within the WHO GISRS: FluNet and FluID (23, 24). We extracted weekly virological and epidemiological surveillance data from 167 countries spanning Week 31 of 2009 through Week 30 of 2020. Key variables included weekly ILI case counts, number of specimens tested, and number of influenza-positive specimens. For selected countries and territories, surveillance data from Week 31 to Week 53 of 2020 were also included. Annual population data for each country were sourced from the United Nations World Population Prospects 2022^1^. To align with influenza surveillance periods, population estimates for January 1 and July 1 of each year from 2009 to 2020 were collected.

### Indicator definitions

The ILI was defined according to the WHO global influenza surveillance standards as an acute respiratory infection with measured fever (≥38 °C), cough, and onset within the preceding 10 days. Three surveillance indicators were calculated for each influenza transmission zone on a weekly basis:

1. Weekly ILI consultation rate (ILI rate): the sum of reported ILI cases across countries within a transmission zone divided by the sum of the populations of reporting countries. For transmission zones with epidemic seasons defined as Week 31 to Week 30 of the following year, population data from January 1 of the following year were used; for zones with epidemic seasons spanning Week 1 to Week 52/53, population data from July 1 of the corresponding year were applied.
2. Weekly influenza virus positivity rate (IV Positivity rate): the sum of influenza-positive specimens across countries within a transmission zone divided by the sum of specimens tested in those countries.
3. Weekly influenza incidence: estimated as the product of the ILI rate and the IV positivity rate, consistent with previous studies (25).

### Global influenza transmission zones

The WHO classification of global influenza transmission zones, published in 2018, groups countries and territories into 18 zones based on shared influenza transmission patterns. This zonal framework was adopted as the geographical unit for all subsequent analyses. The 18 transmission zones are illustrated in Supplementary Figure 1.

### MEM model construction

The MEM model was used to establish epidemic intensity thresholds for each transmission zone and surveillance indicator. MEM requires a minimum of five consecutive influenza seasons of weekly surveillance data to produce stable threshold estimates, with an upper limit of 10 seasons recommended to avoid over-smoothing (16, 22). To avoid confounding effects of the 2009 influenza pandemic and the COVID-19 pandemic and its associated non-pharmaceutical interventions, the 2009/10 season, the 2019/20 season and subsequent seasons were excluded. The final modeling dataset comprised the 2010/11 through 2018/19 seasons for most zones; for certain Southern Hemisphere and tropical zones, seasons from 2011 to 2019 were used. To further evaluate threshold performance in regular seasons, we removed the 2018/19 or 2019 season from each transmission zone and refitted the MEM models, the resulting thresholds are detailed in Supplementary Tables 1, 2. To handle missing weekly values in the historical surveillance data, we applied the data transformation function implemented in the MEM R package, a standard preprocessing step in MEM modeling that is recommended to stabilize season-to-season variability while preserving the underlying epidemic pattern. Missing values are imputed using a linear interpolation between the two neighboring non-missing values; if the first or last value is missing, it is imputed using the closest non-missing value (constant interpolation). Given that such isolated missing values were very limited across the included transmission zones, the impact of imputation on model estimation and threshold derivation is expected to be limited.

### Early warning evaluation

For each transmission zone and surveillance indicator, MEM-derived epidemic thresholds were applied to target seasons to evaluate early warning performance. Target seasons included the 2018/19 (or 2019) regular season, the 2019/20 (or 2020) COVID-19 pandemic season, and the 2009/10 (or 2010) influenza pandemic season. MEM separately models each target season to determine the start and end weeks of the pre-epidemic, epidemic, and post-epidemic phases (16). Early warning performance was assessed using the following metrics:

Alert week: the first week of the target season during which the indicator value exceeded the epidemic threshold.

Early warning: weeks by which the alert week preceded the epidemic start week.

Delayed warning: weeks by which the alert week lagged behind the epidemic start week.

Paired *t*-tests were used to compare alert weeks and epidemic start weeks across all target seasons for each indicator. The early warning performance of the three indicators was compared cross-sectionally, using the IV positivity rate as the reference given its established specificity and stability.

### Peak timing simulation

For each indicator within each transmission zone, the alert week was first identified for every historical season using the MEM-derived epidemic threshold. For each historical season, the interval (in weeks) between the alert week and the observed epidemic peak week was calculated (26). The mean interval across all historical seasons was then computed and retained to two decimal places.

**Figure 1:**
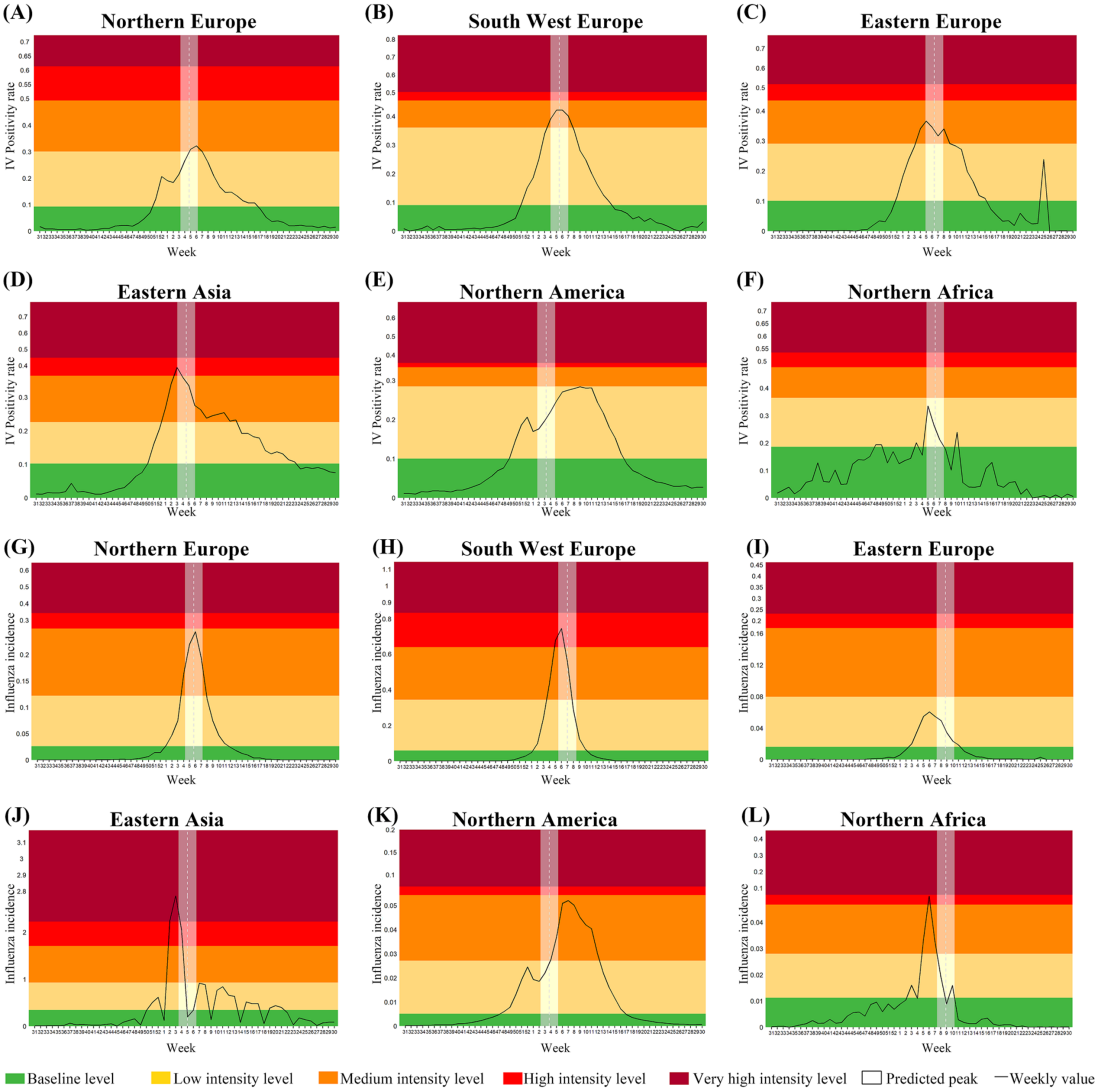
Peak timing simulation results for IV positivity rate and influenza incidence across influenza transmission zones. Labels **(A-L)** display the results of different influenza transmission zones.

To simulate the expected peak week for a target season, the alert week of that target season was identified, and the mean historical interval was added. The performance of peak timing simulation was evaluated by comparing the simulated peak week with the observed peak week for each target season. Paired t-tests were used to assess the overall agreement between simulated and observed peak weeks across all target seasons. For visual display purposes only, we shaded the expected peak week ± 1.5 weeks in Figure 1 to illustrate the proximity between simulated and observed peaks.

### Sensitivity analysis

The accuracy of each MEM model was assessed using a leave-one-out cross-validation procedure. For each transmission zone, each influenza season in the modeling dataset was iteratively designated as the target season, with the remaining seasons used to re-estimate the MEM model and its epidemic threshold. The recalculated threshold was then compared against the actual epidemic and non-epidemic periods of the target season. Sensitivity, specificity, positive predictive value, negative predictive value, and Youden’s index were calculated for each iteration. This process was repeated for all seasons included in the model.

All statistical analyses and MEM modeling were performed using R version 4.3.2 (R Foundation for Statistical Computing). Maps of global influenza transmission zones were generated using ArcMap software (version 10.2). All tests were two-sided, with *p* < 0.05 considered statistically significant.

## Results

### Global surveillance overview

Between Week 31 of 2009 and Week 30 of 2020, a total of 135,138,758 ILI cases were reported across 167 countries within GISRS. The mean ILI rate was 0.2062 per 100,000 population. Of 27,930,197 specimens tested, 4,866,938 were positive for influenza, yielding a mean IV positivity rate of 17.43%. The mean influenza incidence was 0.0359 per 100,000 population (Table 1).

**Table 1:** Overview of influenza surveillance data across global transmission zones, 2009–2020.

ITZ	Sample processed	IV positive cases	IV positive rate (%)	ILI cases	ILI rate (per 100,000)	Influenza incidence (per 100,000)	Number of countries*
Central America and Caribbean	633,666	155,388	24.52	338,055	0.0432	0.0067	25
Central Asia	60,131	8,437	14.03	236,468	0.0116	0.0010	5
Eastern Africa	146,419	23,486	16.04	250,010	0.0045	0.0006	10
Eastern Asia	5,124,126	745,414	14.55	2,236,644	2.4250	0.3704	5
Eastern Europe	1,662,941	294,995	17.74	11,996,247	0.0883	0.0152	11
Middle Africa	48,611	6,599	13.58	86,922	0.0037	0.0005	5
North America	12,049,648	1,966,331	16.32	10,074,994	0.0546	0.0091	3
Northern Africa	166,154	37,084	22.32	921,570	0.0490	0.0109	5
Northern Europe	1,408,757	495,803	35.19	1,408,757	0.0760	0.0166	13
Oceania Melanesia Polynesia	490,021	63,165	12.89	791,542	0.0545	0.0068	5
South West Europe	2,414,769	548,484	22.71	19,555,767	0.1433	0.0422	19
South-East Asia	321,461	67,753	21.08	38,052,998	0.5691	0.1048	10
Temperate South America	1,364,588	88,683	6.50	9,030,779	0.3638	0.0224	4
Tropical South America	642,060	78,429	12.22	15,453,235	0.2456	0.0440	9
Western Africa	162,262	24,544	15.13	218,691	0.0025	0.0003	11
Western Asia	618,960	146,898	23.73	24,241,807	0.4533	0.0775	17
Southern Africa	84,000	13,090	15.58	5,581	0.0006	0.0001	1
Southern Asia	531,623	102,355	19.25	238,691	0.0115	0.0020	9
Globe	27,930,197	4,866,938	17.43	135,138,758	0.2062	0.0359	167

* indicates the number of countries reporting influenza surveillance data within each transmission zone.

The distribution of surveillance activity varied substantially across transmission zones. The three zones reporting the highest ILI case counts were Southeast Asia (38,052,998), West Asia (24,241,807), and Southwestern Europe (19,555,767), whereas South Africa reported the lowest (5,581). The highest numbers of influenza-positive specimens were detected in North America (1,966,331), East Asia (745,414), and Eastern Europe (294,995), with the lowest in South Africa (13,090). The highest mean IV positivity rates were observed in Northern Europe (35.19%), West Asia (23.73%), and Central America and the Caribbean (24.52%), while Temperate South America recorded the lowest (6.50%). The highest mean ILI rates and influenza incidence were both found in Southeast Asia (0.5691 and 0.1048 per 100,000, respectively), West Asia (0.4533 and 0.0775 per 100,000), and Temperate South America (0.3638 and 0.0224 per 100,000), whereas South Africa had the lowest values (0.0006 and 0.0001 per 100,000) (Table 1 and Supplementary Figure 2).

### Epidemic patterns and threshold systems

**Figure 2:**
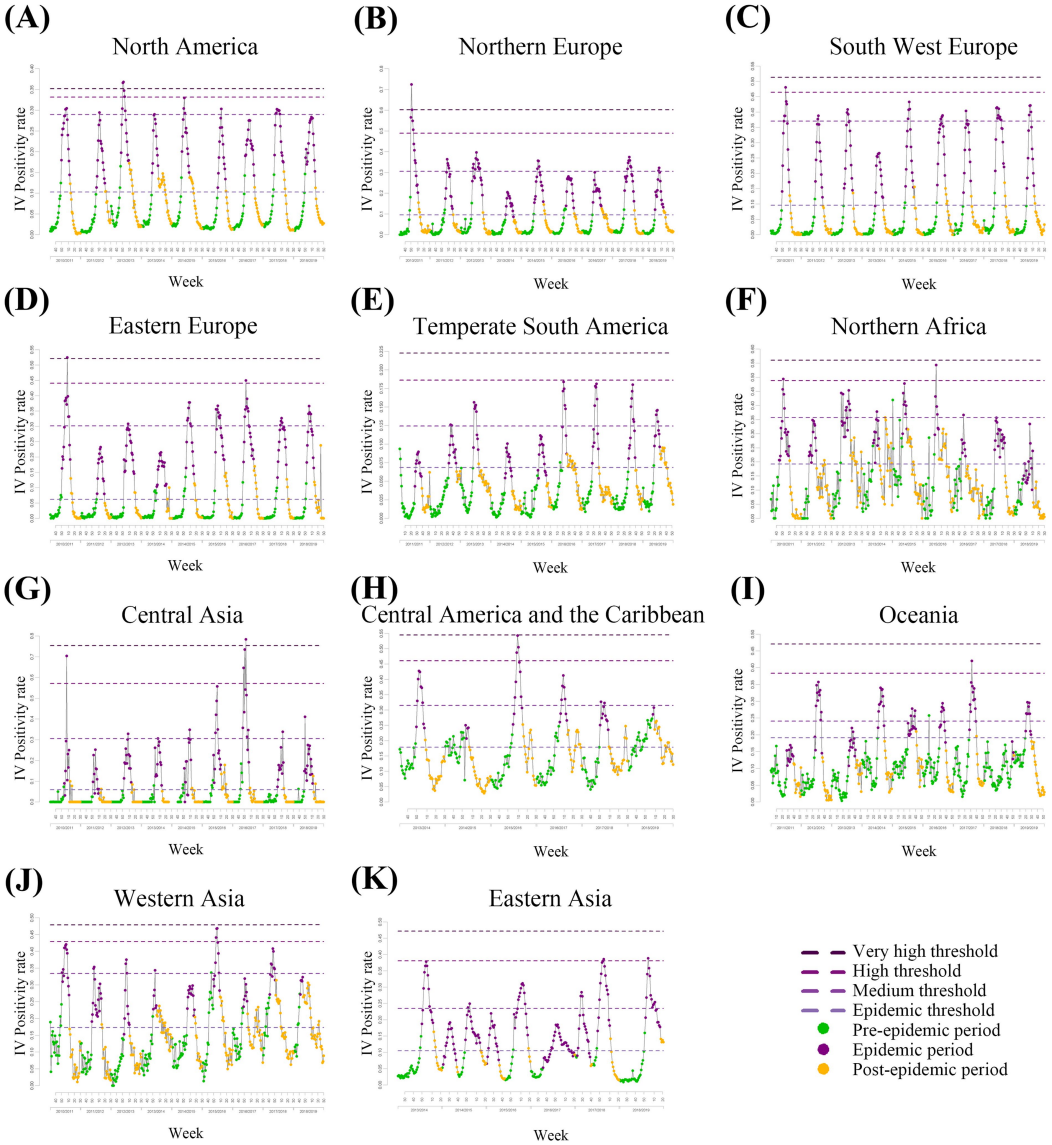
Influenza epidemic patterns and MEM model fitting in global transmission zones with seasonal single-peak and dual-peak epidemics. Labels **(A-K)** display the results of different influenza transmission zones.

Three distinct epidemic patterns were identified across the 18 transmission zones. In North America, Northern Europe, Southwestern Europe, Eastern Europe, and Temperate South America, all three indicators, ILI rate, IV positivity rate, and influenza incidence, exhibited a clear seasonal single-peak pattern (Figure 2). Single wave models were fitted for each zone and indicator. In North America, the four-level intensity thresholds for the IV positivity rate were 10.30% (epidemic), 28.96% (medium), 33.15% (high), and 35.19% (very high). In Northern Europe, the corresponding thresholds were 9.58, 30.52, 48.90, and 60.24%; in Southwestern Europe, they were 9.58, 37.03, 46.44, and 51.33%. The threshold system for other transmission zones is shown in Supplementary Table 1.

In East Asia, the IV positivity rate displayed both single-peak and dual-peak characteristics (Figure 2K). Both single wave and double wave models were fitted and compared. The single wave model yielded an epidemic threshold of 22.48%, whereas the double wave model yielded a lower threshold of 10.49%, providing higher sensitivity for early detection of epidemic onset. Consequently, the double wave model was selected for the IV positivity rate in East Asia, with the four-level intensity thresholds determined as 10.49, 23.46, 38.08, and 47.16%. The ILI rate and influenza incidence in this zone both exhibited single-peak patterns and were modeled accordingly (Supplementary Table 1).

In most African and tropical zones, including Southeast Asia, Tropical South America, East Africa, Central Africa, and West Africa, all three indicators demonstrated year-round or irregular epidemic patterns. Single wave and double wave models were compared for these zones. The double wave model markedly prolonged the epidemic phase by incorporating extended low-activity troughs, substantially reducing specificity and limiting its utility for outbreak detection against a background of year-round fluctuation. We therefore adopted intensity thresholds derived from the single wave model for these zones. The full threshold system for all transmission zones is provided in Supplementary Table 1.

### Early warning performance

**Figure 3:**
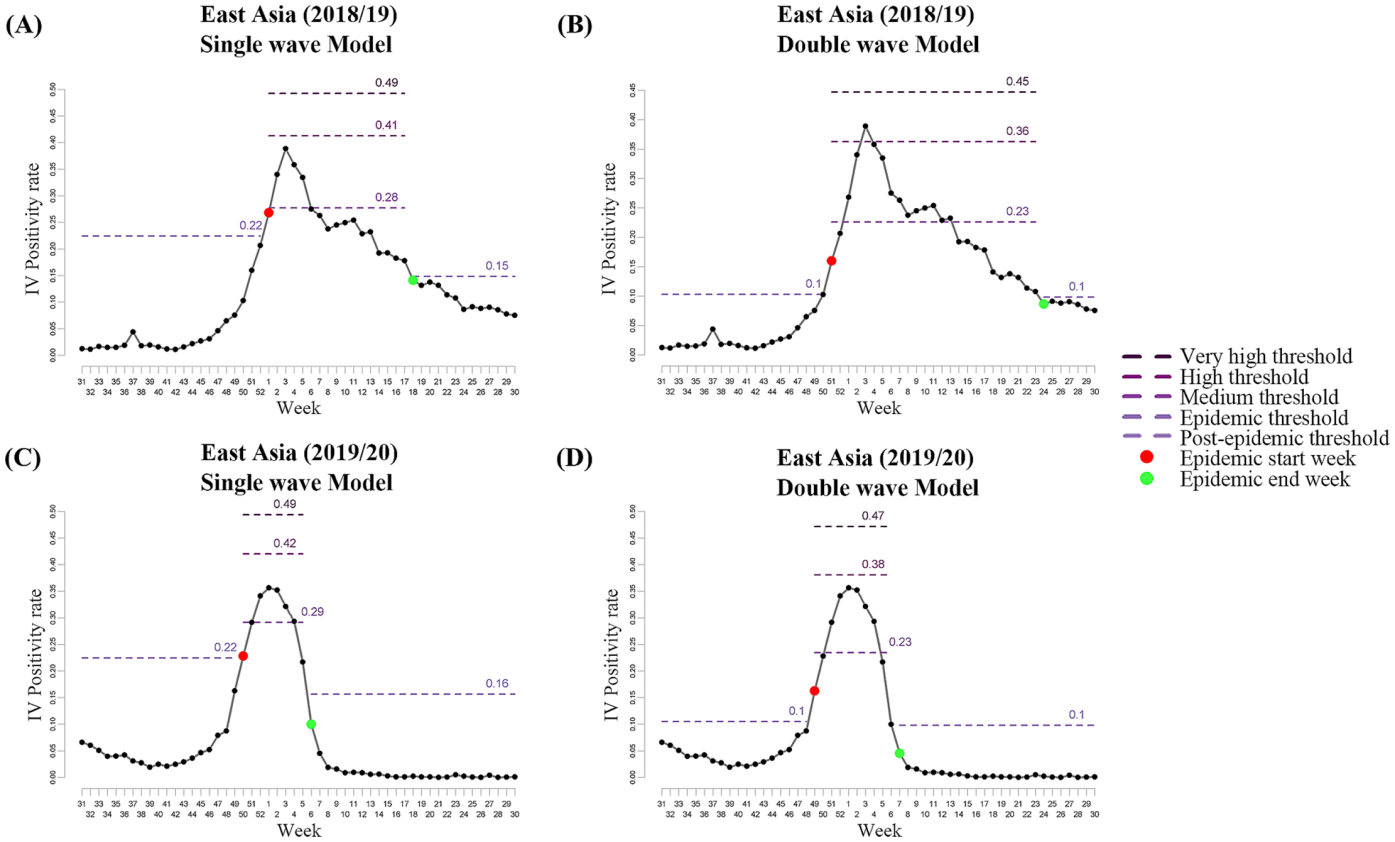
Comparison of early warning performance between single wave and double wave models in the East Asia transmission zone. **(A)** Single wave model, 2018/19 season. **(B)** Double wave model, 2018/19 season. **(C)** Single wave model, 2019/20 season. **(D)** Double wave model, 2019/20 season.

The IV positivity rate demonstrated the most robust early warning performance, with timely alert across the majority of transmission zones (Table 2). Alert-to-epidemic-start differences were within ±2 weeks in most zones. Exact concordance between the alert week and epidemic start week occurred 23 times, predominantly in Eastern Europe, North America, and Central Africa. A difference of ±1 week occurred 12 times, including 3 instances of early warning (Central America and the Caribbean, East Asia, Southwestern Europe) and 9 instances of delayed warning (mainly in Northern Europe and East Africa). A difference of ±2 weeks occurred 4 times, with 1 early warning (Southwestern Europe) and 3 delayed warnings (North Africa, Temperate South America, Central America and the Caribbean). In East Asia, the double wave model for IV positivity rate achieved early warning of 1–2 weeks, outperforming the single wave model (Figure 3). Overall, no statistically significant difference was observed between alert weeks and epidemic start weeks across all zones (*t* = −0.23, *p* = 0.82; mean difference = −0.11 weeks, 95% CI: −1.13 to 0.90).

**Table 2:** Early warning performance of each surveillance indicator across global transmission zones in target seasons.

ITZ	2018/19 (2019)			2019/20 (2020)			2009/10 (2010)		
	IV positive rate	ILI rate	Incidence	IV positive rate	ILI rate	Incidence	IV positive rate	ILI rate	Incidence
Central America and Caribbean	−1	–	–	2	–	–	0	–	–
Central Asia	0	–	1	0	–	0	−8	–	0
Eastern Africa	1	1	−22	1	−17	1	0	–	–
Eastern Asia	−1	0	−3	0	−3	0	–	0	0
Eastern Europe	0	–	1	0	–	1	0	0	−4
Middle Africa	0	–	–	0	–	–	0	–	–
North America	0	−4	−1	0	−6	−3	0	0	0
Northern Africa	2	1	2	1	–	2	8	−4	−4
Northern Europe	0	0	−1	1	1	4	1	–	3
Oceania Melanesia Polynesia	0	–	−5	–	–	–	7	–	2
South West Europe	−1	0	0	1	1	1	−2	3	1
South-East Asia	–	–	–	0	–	–	−16	–	–
Temperate South America	0	0	1	–	–	–	2	−1	−1
Tropical South America	0	–	–	0	–	–	1	–	–
Western Africa	4	–	–	1	–	–	0	–	–
Western Asia	0	–	–	1	–	–	0	–	–

Values indicate the difference (in weeks) between the alert week and the epidemic start week. Negative values denote early warning (alert precedes epidemic start), positive values denote delayed warning (alert follows epidemic start), and 0 denotes exact concordance. Hyphens (–) indicate no observations due to insufficient data for that specific indicator.

Early warning performance of the ILI rate was slightly inferior to that of the IV positivity rate, although differences remained within ±3 weeks in most zones (Table 2). Exact concordance occurred 7 times, and a difference of ±1 week occurred 5 times (1 early warning, 4 delayed warnings). Across all zones, no significant difference was found between alert weeks and epidemic start weeks (*t* = 1.29, *p* = 0.21; mean difference = 1.32 weeks, 95% CI: −0.83 to 3.46).

The influenza incidence showed less stable early warning performance compared with the IV positivity rate. While alert-to-epidemic-start differences were within ±2 weeks in most zones, substantial variability was observed in several regions (Table 2). Exact concordance occurred 6 times (East Asia, Central Asia, North America, Southwestern Europe). A difference of ±1 week occurred 10 times (3 early warnings in North America, Northern Europe, and Temperate South America; 7 delayed warnings mainly in Eastern Europe, Southwestern Europe, Temperate South America, and East Africa). Across all zones, no significant difference was observed (*t* = 0.97, *p* = 0.34; mean difference = 0.89 weeks, 95% CI: −0.99 to 2.76).

Cross-indicator comparison with the IV positivity rate as the reference showed that the ILI rate achieved earlier warning on 6 occasions, whereas the influenza incidence achieved earlier warning on 4 occasions; all remaining observations showed simultaneous or delayed warnings relative to the IV positivity rate. In summary, the IV positivity rate exhibited the best combination of stability and specificity globally. The ILI rate and influenza incidence performed adequately in zones with high-quality surveillance data (e.g., Europe and North America) but poorly in African and tropical zones.

### Peak timing simulation performance

The IV positivity rate provided the broadest coverage across transmission zones, with 30 evaluable observations. The simulated-to-observed peak difference was within ±3 weeks for 16 observations, concentrated in East Asia, Europe, Northern America, and Northern Africa (Figure 1). In zones with highly variable epidemic patterns, such as Southeast Asia, Tropical South America, and Central America and the Caribbean, simulation errors generally exceeded 10 weeks (Supplementary Table 3). No significant difference was found between simulated and observed peak weeks (*t* = −0.01, *p* = 0.99; mean difference = −0.02 weeks, 95% CI: −3.36 to 3.33).

Among the three indicators, influenza incidence achieved the best peak simulation accuracy. Of 18 evaluable observations, 11 had simulation errors within ±3 weeks, including Southwestern Europe (1.0, 1.8 weeks), Northern Europe (0.25, 1.2 weeks), North Africa (2.56, 2.88 weeks), Eastern Europe (2.75, 2.8 weeks), East Asia (1.5, 2.0 weeks), and Temperate South America (2.57 weeks). Simulation performance was poorer in Central Asia, East Africa, and Oceania (Supplementary Table 3). No significant difference was observed between simulated and observed peak weeks (*t* = 0.15, *p* = 0.87; mean difference = 0.21 weeks, 95% CI: −2.62 to 3.05).

The ILI rate showed the weakest peak simulation performance. Of 13 evaluable observations, only 5 had simulation errors within ±3 weeks, including North Africa, Southwestern Europe, Northern Europe, and East Asia. Differences exceeded 9 weeks in North America and 10 weeks in East Africa and Temperate South America (Supplementary Table 3). No significant difference was observed (*t* = 0.01, *p* = 0.99; mean difference = 0.03 weeks, 95% CI: −5.05 to 5.10).

### Sensitivity analysis

The sensitivity and specificity of MEM models were generally high in zones with seasonal single-peak or dual-peak patterns. In Northern Europe, Southwestern Europe, North Africa, and North America, sensitivity and specificity both exceeded 75% across all three indicators, with most values above 90%. In Eastern Europe, East Asia, and Temperate South America, the IV positivity rate and influenza incidence achieved sensitivity and specificity above 80%, whereas the ILI rate yielded values above 60%. In Central Asia, both the IV positivity rate and influenza incidence exceeded 90%. In East Africa, the ILI rate and influenza incidence outperformed the IV positivity rate.

Overall, model performance was strongest in zones with high-quality surveillance data and clear seasonal patterns (e.g., Northern Europe, Southwestern Europe, East Asia) and weakest in tropical and African zones with year-round or irregular activity. Across indicators, the IV positivity rate and influenza incidence generally outperformed the ILI rate (Supplementary Table 4).

## Discussion

This study established a comprehensive, region-specific epidemic intensity threshold system for influenza across global WHO transmission zones based on their specific epidemic patterns, and systematically evaluated the performance of three surveillance indicators, ILI rate, IV positivity rate, and influenza incidence, in early warning and peak timing simulation. Overall, the IV positivity rate demonstrated the best stability, specificity, and geographic coverage across the three surveillance indicators, making it the most reliable indicator for epidemic threshold construction globally. The ILI rate and influenza incidence performed well in zones with high-quality surveillance data and distinct seasonal patterns, such as Europe and North America, but showed limited utility in tropical and African zones with year-round or irregular epidemic activity. These findings underscore the necessity of tailoring epidemic thresholds to local transmission patterns rather than adopting a uniform criterion, and highlight the differential applicability of virological, syndromic, and composite indicators across diverse epidemiological settings.

The marked heterogeneity of influenza epidemic patterns, ranging from predictable seasonal single peaks in temperate regions to year-round or irregular activity in much of the tropics and Africa, reinforces the argument against a one-size-fits-all epidemic threshold. Some settings have empirically applied a 10% positivity threshold to define epidemic onset (16, 17), yet the optimal thresholds derived in this study varied considerably across transmission zones depending on local epidemic characteristics and surveillance intensity. A globally uniform threshold would inevitably misclassify epidemic periods in numerous zones, delaying or falsely triggering alerts. Developing region-specific thresholds is therefore essential not only for accurate epidemic intensity assessment within each zone but also for meaningful cross-zone comparisons of influenza activity.

The MEM framework, recommended by ECDC and WHO (17–19), proved effective across zones with distinct seasonal patterns. In temperate and subtropical zones exhibiting single or dual seasonal peaks, model sensitivity and specificity both exceeded 80% for virological and composite indicators, consistent with previous validations in Europe and North America (16, 18, 20–22). The East Asia zone presented a unique challenge due to its mixed single- and dual-peak virological pattern; the double wave model yielded a substantially lower epidemic threshold and achieved 1–2 weeks of early warning, demonstrating the importance of aligning model structure with local epidemic periodicity. In contrast, for zones with year-round or irregular patterns, the double wave model excessively extended the epidemic phase into low-activity troughs, severely compromising specificity. The single wave model, by confining the epidemic phase to periods of elevated activity, offered greater practical value for outbreak detection in these settings.

Among the three indicators evaluated, the IV positivity rate demonstrated the best overall stability, specificity, and geographic coverage. Virological surveillance data benefited from greater continuity and completeness across zones compared with syndromic ILI data, which are subject to heterogeneity in case definitions, reporting practices, and healthcare access (10, 17, 27). The influenza incidence, a composite of ILI rate and IV positivity rate, achieved the highest peak timing simulation accuracy in zones with adequate data quality, suggesting that combining epidemiological and virological information can improve certain predictive tasks (28). However, both ILI rate and influenza incidence performed poorly in African and tropical zones, where syndromic surveillance infrastructure remains underdeveloped. The limited early warning performance of ILI rate in tropical and subtropical settings corroborates previous observations that ILI-based thresholds are less reliable in these regions (29–32). Beyond the syndromic surveillance challenges, the IV positivity rate in these zones, while demonstrating better continuity than ILI-based indicators, was also constrained by persistently low specimen collection and testing density and a high proportion of low positive case counts, making it difficult to establish stable estimates that reliably reflect true epidemic dynamics. Moreover, the year-round or irregular influenza activity that characterizes most tropical and African settings presents a more fundamental challenge: the MEM framework, which relies on the assumption of well-defined seasonal epidemic periods, is inherently less suited to capturing predictable epidemic timing in these contexts. Consequently, the threshold systems derived for these zones are better suited for detecting aberrant surges, such as pandemic or outbreak signals, rather than for predicting routine seasonal peaks. This distinction underscores the urgent need for developing surveillance and early warning approaches specifically tailored to the unique epidemiological characteristics of tropical and irregular-epidemic settings.

Several limitations should be acknowledged. First, data heterogeneity across countries within each transmission zone represents a notable concern. Surveillance capacity, ILI case definitions, specimen collection density, and laboratory testing capability vary markedly across countries pooled within each zone (17). Zone-level aggregation may average the epidemic levels of individual countries within each zone, potentially producing thresholds that underestimate the actual levels in high-intensity countries while overestimating those in low-intensity ones. Nevertheless, our aggregation followed the WHO classification of influenza transmission zones, which groups countries with broadly similar influenza transmission patterns, so the impact of such pooling on the underlying epidemic dynamics at the zonal level is limited, and the resulting thresholds can still provide useful early warning and peak timing references at zone scales. However, due to data sparsity at the country level and space constraints, we did not conduct country-level MEM modeling or sensitivity analyses. We therefore emphasize that the thresholds derived in this study should be interpreted as zone-level benchmarks rather than country-level prescriptive criteria, and their application at national scales should be calibrated against local surveillance characteristics in future research. Second, to avoid confounding by the COVID-19 pandemic and associated non-pharmaceutical interventions while maintaining the minimum five-season requirement of MEM, the 2019/20 season and subsequent seasons were excluded. Third, in zones with severely limited surveillance data, such as South Africa and South Asia, historical data were too sparse and of insufficient quality to support in-depth analysis; consequently, results were available for only 16 of the 18 transmission zones. Furthermore, in zones such as Central Africa, West Africa, and West Asia, only virological surveillance data could be analyzed, and the reliability of trend and intensity estimates in these settings may be further constrained by persistently low weekly positive case counts and the absence of well-defined seasonal patterns. Fourth, the inclusion of the 2009/10 pandemic season as a target for early warning evaluation introduces an inherent asymmetry, as the models were trained on post-2010 seasonal data and the pandemic represents a fundamentally different transmission dynamic. Results from this season should therefore be interpreted with caution, and early warning in this context should be understood as the detection of aberrant activity exceeding background levels rather than the prediction of seasonal epidemic timing. Despite these limitations, this study provides the first systematic, globally comparative assessment of MEM-based threshold systems across WHO transmission zones using a consistent analytical framework. The resulting threshold system offers a practical tool for calibrating influenza early warning and peak timing prediction to local epidemic characteristics, and provides a scientific basis for strengthening region-specific influenza prevention and control strategies.

## Conclusion

This study systematically analyzed influenza epidemic patterns across global WHO transmission zones from 2009 to 2020. Three distinct epidemic patterns were identified: typical seasonal single-peak patterns in temperate zones like North America, Europe, and Temperate South America; mixed single- and dual-peak patterns in East Asia; and year-round or irregular patterns in most African and tropical zones. Accordingly, zone-specific models were fitted to establish a comprehensive, region-specific epidemic intensity threshold system. Based on this threshold system, the early warning and peak timing simulation strategies achieved reliable performance in zones with well-defined seasonal patterns, with sensitivity and specificity exceeding 80%, early warning within ±2 weeks for the IV positivity rate, and peak timing errors within ±3 weeks in the majority of evaluable observations. In tropical and African zones with year-round or irregular activity, however, performance was more limited, and the threshold systems in these settings are better suited for detecting aberrant surges than for predicting routine seasonal peaks. Among the three surveillance indicators evaluated, the IV positivity rate exhibited the best stability, specificity, and geographic coverage, while the ILI rate and influenza incidence performed adequately in zones with high-quality data and distinct seasonality. These findings provide a scientific basis for calibrating influenza early warning and peak prediction to local epidemic characteristics and for strengthening region-specific prevention and control strategies globally.

## Data Availability

Publicly available datasets were analyzed in this study. This data can be found: https://www.who.int/initiatives/global-influenza-surveillance-and-response-system.

## Footnotes

1
https://population.un.org/wpp/
