# Supplementary material for: Global region-specific influenza early warning by establishing an epidemic intensity threshold system: a modeling study
Source: Front Public Health. 2026 Aug 4;14:1885724. doi: 10.3389/fpubh.2026.1885724 (PMC13481509; doi:10.3389/fpubh.2026.1885724)
Supplement: Supplementary file 1 [file Supplementary_file_1.DOCX]

**Supplementary Table legend**

Supplementary Table 1. Epidemic intensity threshold systems for each surveillance indicator across global influenza transmission zones.

| **ITZ** | **Indicator** | **Epidemic threshold** | **Medium threshold** | **High threshold** | **Very high threshold** |
| --- | --- | --- | --- | --- | --- |
| **Central America and Caribbean** | IV Positivity rate (%) | 17.87 | 31.48 | 46.05 | 54.48 |
| **Central Asia** | IV Positivity rate (%) | 5.91 | 30.57 | 57.16 | 75.38 |
|  | Influenza incidence | 0.0007 | 0.0048 | 0.0090 | 0.0119 |
| **Eastern Africa** | IV Positivity rate (%) | 21.60 | 28.23 | 43.57 | 52.78 |
|  | ILI rate | 0.0042 | 0.0061 | 0.0095 | 0.0117 |
|  | Influenza incidence | 0.0013 | 0.0011 | 0.0018 | 0.0022 |
| **Eastern Asia** | IV Positivity rate (%) | 10.49 | 23.46 | 38.08 | 47.16 |
|  | ILI rate | 2.7303 | 4.3007 | 6.4289 | 7.6791 |
|  | Influenza incidence | 0.4104 | 1.0447 | 1.9542 | 2.5773 |
| **Eastern Europe** | IV Positivity rate (%) | 6.15 | 30.15 | 44.05 | 52.08 |
|  | ILI rate | 0.1840 | 0.2381 | 0.4897 | 0.6734 |
|  | Influenza incidence | 0.0118 | 0.0781 | 0.1579 | 0.2156 |
| **Middle Africa** | IV Positivity rate (%) | 12.11 | 29.73 | 45.21 | 54.41 |
| **North America** | IV Positivity rate (%) | 10.30 | 28.96 | 33.15 | 35.19 |
|  | ILI rate | 0.0434 | 0.1036 | 0.1952 | 0.2582 |
|  | Influenza incidence | 0.0072 | 0.0299 | 0.0594 | 0.0804 |
| **Northern Africa** | IV Positivity rate (%) | 19.23 | 35.70 | 48.82 | 56.06 |
|  | ILI rate | 0.0465 | 0.0975 | 0.1557 | 0.1915 |
|  | Influenza incidence | 0.0087 | 0.0298 | 0.0494 | 0.0617 |
| **Northern Europe** | IV Positivity rate (%) | 9.58 | 30.52 | 48.90 | 60.24 |
|  | ILI rate | 0.1341 | 0.5019 | 0.7935 | 0.9716 |
|  | Influenza incidence | 0.0258 | 0.1388 | 0.2701 | 0.3624 |
| **Oceania Melanesia** | IV Positivity rate (%) | 19.14 | 24.06 | 38.31 | 47.05 |
|  | Influenza incidence | 0.0107 | 0.0148 | 0.0390 | 0.0600 |
| **South West Europe** | IV Positivity rate (%) | 9.58 | 37.03 | 46.44 | 51.33 |
|  | ILI rate | 0.2672 | 1.0913 | 1.7131 | 2.0909 |
|  | Influenza incidence | 0.0587 | 0.4074 | 0.6906 | 0.8720 |
| **South-East Asia** | IV Positivity rate (%) | 24.19 | 28.87 | 37.18 | 41.58 |
| **Temperate South America** | IV Positivity rate (%) | 6.85 | 12.44 | 18.62 | 22.26 |
|  | ILI rate | 0.3430 | 0.6255 | 1.0247 | 1.2746 |
|  | Influenza incidence | 0.0243 | 0.0821 | 0.1497 | 0.1953 |
| **Tropical South America** | IV Positivity rate (%) | 12.99 | 24.43 | 39.25 | 48.41 |
| **Western Africa** | IV Positivity rate (%) | 18.14 | 27.40 | 39.58 | 46.56 |
| **Western Asia** | IV Positivity rate (%) | 17.28 | 33.38 | 42.86 | 47.87 |

*Note: Units for ILI rate and influenza incidence are per 100,000 population.*

Supplementary Table 2. Epidemic intensity threshold systems for each surveillance indicator across global influenza transmission zones (excluding the regular epidemic season 2018/19 or 2019).

| **ITZ** | **Indicator** | **Epidemic threshold** | **Medium threshold** | **High threshold** | **Very high threshold** |
| --- | --- | --- | --- | --- | --- |
| **Central America and Caribbean** | IV Positivity rate (%) | 15.33 | 31.69 | 46.86 | 55.71 |
| **Central Asia** | IV Positivity rate (%) | 5.17 | 28.68 | 55.38 | 74.07 |
|  | Influenza incidence | 0.0005 | 0.0043 | 0.0093 | 0.0132 |
| **Eastern Africa** | IV Positivity rate (%) | 19.32 | 27.88 | 43.56 | 53.05 |
|  | ILI rate | 0.0042 | 0.0059 | 0.0091 | 0.0111 |
|  | Influenza incidence | 0.0011 | 0.0011 | 0.0018 | 0.0023 |
| **Eastern Asia** | IV Positivity rate (%) | 10.16 | 22.55 | 36.32 | 44.83 |
|  | ILI rate | 2.7171 | 4.1018 | 6.0773 | 7.2306 |
|  | Influenza incidence | 0.3446 | 0.9292 | 1.7027 | 2.2253 |
| **Eastern Europe** | IV Positivity rate (%) | 10.06 | 29.07 | 43.39 | 51.79 |
|  | ILI rate | 0.1413 | 0.2536 | 0.5120 | 0.6984 |
|  | Influenza incidence | 0.0160 | 0.0795 | 0.1667 | 0.2313 |
| **Middle Africa** | IV Positivity rate (%) | 11.35 | 27.54 | 43.14 | 52.60 |
| **North America** | IV Positivity rate (%) | 9.99 | 28.42 | 33.30 | 35.71 |
|  | ILI rate | 0.0455 | 0.0956 | 0.1752 | 0.2290 |
|  | Influenza incidence | 0.0051 | 0.0271 | 0.0543 | 0.0737 |
| **Northern Africa** | IV Positivity rate (%) | 18.59 | 36.42 | 47.54 | 53.48 |
|  | ILI rate | 0.0479 | 0.0911 | 0.1395 | 0.1684 |
|  | Influenza incidence | 0.0112 | 0.0280 | 0.0467 | 0.0586 |
| **Northern Europe** | IV Positivity rate (%) | 9.25 | 30.02 | 49.26 | 61.32 |
|  | ILI rate | 0.1277 | 0.4432 | 0.7507 | 0.9476 |
|  | Influenza incidence | 0.0260 | 0.1218 | 0.2493 | 0.3421 |
| **Oceania Melanesia** | IV Positivity rate (%) | 18.34 | 23.17 | 37.91 | 47.11 |
|  | Influenza incidence | 0.0119 | 0.0130 | 0.0340 | 0.0520 |
| **South West Europe** | IV Positivity rate (%) | 8.99 | 36.03 | 45.41 | 50.30 |
|  | ILI rate | 0.2713 | 0.9660 | 1.5900 | 1.9816 |
|  | Influenza incidence | 0.0584 | 0.3439 | 0.6375 | 0.8374 |
| **South-East Asia** | IV Positivity rate (%) | 23.21 | 27.88 | 35.86 | 40.07 |
| **Temperate South America** | IV Positivity rate (%) | 6.59 | 11.97 | 18.35 | 22.18 |
|  | ILI rate | 0.3475 | 0.6694 | 1.0542 | 1.2885 |
|  | Influenza incidence | 0.0262 | 0.0873 | 0.1571 | 0.2036 |
| **Tropical South America** | IV Positivity rate (%) | 13.23 | 24.40 | 40.42 | 50.52 |
| **Western Africa** | IV Positivity rate (%) | 18.33 | 26.29 | 37.96 | 44.65 |
| **Western Asia** | IV Positivity rate (%) | 17.46 | 32.71 | 43.29 | 49.00 |

*Note: Units for ILI rate and influenza incidence are per 100,000 population.*

Supplementary Table 3. Peak timing simulation performance of each surveillance indicator across global transmission zones in target seasons.

| **ITZ** | **2018/19 （2019）** | | | **2019/20 （2020）** | | |
| --- | --- | --- | --- | --- | --- | --- |
|  | **IV Positive rate** | **ILI rate** | **Incidence** | **IV Positive rate** | **ILI rate** | **Incidence** |
| **Central America and Caribbean** | -12.20 | — | — | -5.17 | — | — |
| **Central Asia** | -2.88 | — | 7.63 | 3.11 | — | -4.11 |
| **Eastern Africa** | 11.63 | 15.86 | 9.40 | -9.38 | -19.00 | -15.00 |
| **Eastern Asia** | 1.56 | 3.67 | 2.00 | 1.40 | -2.25 | -1.50 |
| **Eastern Europe** | 1.38 | — | 2.75 | 0.11 | — | 2.80 |
| **Middle Africa** | 2.63 | — | — | -2.89 | — | — |
| **North America** | -5.75 | -9.50 | -3.25 | -4.22 | -7.78 | -4.78 |
| **Northern Africa** | 1.25 | 0.75 | 2.88 | 5.11 | 5.67 | 2.56 |
| **Northern Europe** | -1.25 | 1.50 | -0.25 | 1.78 | -3.20 | -1.20 |
| **Oceania Melanesia Polynesia** | 3.83 | — | -9.14 | — | — | — |
| **South West Europe** | -0.50 | 1.25 | 1.00 | -0.89 | 1.00 | 1.80 |
| **South-East Asia** | — | — | — | 18.00 | — | — |
| **Temperate South America** | 0.13 | 13.14 | 2.57 | — | — | — |
| **Tropical South America** | 16.14 | — | — | 5.25 | — | — |
| **Western Africa** | 7.88 | — | — | 1.00 | — | — |
| **Western Asia** | 1.75 | — | — | -5.44 | — | — |

*Note: Values indicate the difference (in weeks) between the simulated peak week and the observed peak week. Negative values denote that the simulated peak preceded the observed peak, positive values denote that the simulated peak lagged behind the observed peak, and 0 denotes exact concordance. Hyphens (—) indicate no observations due to insufficient data for that specific indicator.*

Supplementary Table 4. Sensitivity analysis results of MEM models across global influenza transmission zones.

| **ITZ** | **Indicator** | **Sensitivity (%)** | **Specificity (%)** | **Positive predictive value (%)** | **Negative predictive value (%)** | **Youden's index (%)** | **Number of seasons included** |
| --- | --- | --- | --- | --- | --- | --- | --- |
| **Central America and Caribbean** | IV Positive rate | 66.62 | 81.15 | 50.32 | 89.46 | 47.77 | 6 |
| **Central Asia** | IV Positive rate | 90.19 | 91.47 | 76.99 | 96.72 | 81.66 | 9 |
|  | Influenza incidence | 90.35 | 92.34 | 76.81 | 97.15 | 82.70 | 9 |
| **Eastern Africa** | IV Positive rate | 35.80 | 91.45 | 46.58 | 87.25 | 27.25 | 9 |
|  | ILI rate | 65.24 | 69.89 | 38.47 | 87.45 | 35.12 | 8 |
|  | Influenza incidence | 44.71 | 86.81 | 45.77 | 86.31 | 31.52 | 8 |
| **Eastern Asia** | IV Positive rate | 86.69 | 82.19 | 82.41 | 86.51 | 68.88 | 6 |
|  | ILI rate | 61.81 | 71.35 | 44.49 | 83.41 | 33.16 | 4 |
|  | Influenza incidence | 83.83 | 86.42 | 69.11 | 93.65 | 70.25 | 4 |
| **Eastern Europe** | IV Positive rate | 96.59 | 87.86 | 76.37 | 98.45 | 84.45 | 9 |
|  | ILI rate | 66.59 | 78.29 | 43.69 | 90.26 | 44.88 | 5 |
|  | Influenza incidence | 95.54 | 90.01 | 73.52 | 98.58 | 85.55 | 5 |
| **Middle Africa** | IV Positive rate | 54.95 | 82.48 | 41.21 | 89.12 | 37.43 | 9 |
| **North America** | IV Positive rate | 94.51 | 84.15 | 69.79 | 97.54 | 78.66 | 9 |
|  | ILI rate | 83.32 | 72.33 | 50.31 | 92.81 | 55.66 | 9 |
|  | Influenza incidence | 92.48 | 88.32 | 72.47 | 97.25 | 80.80 | 9 |
| **Northern Africa** | IV Positive rate | 82.57 | 75.12 | 52.07 | 92.94 | 57.69 | 9 |
|  | ILI rate | 80.59 | 77.05 | 53.99 | 92.23 | 57.64 | 9 |
|  | Influenza incidence | 91.15 | 86.41 | 69.91 | 96.57 | 77.56 | 9 |
| **Northern Europe** | IV Positive rate | 94.24 | 89.54 | 78.06 | 97.52 | 83.78 | 9 |
|  | ILI rate | 89.79 | 92.42 | 78.47 | 96.71 | 82.21 | 5 |
|  | Influenza incidence | 96.77 | 93.52 | 79.82 | 99.09 | 90.30 | 5 |
| **Oceania Melanesia** | IV Positive rate | 74.06 | 82.68 | 52.73 | 92.43 | 56.73 | 9 |
|  | Influenza incidence | 69.18 | 79.92 | 48.29 | 90.54 | 49.10 | 9 |
| **South West Europe** | IV Positive rate | 96.03 | 92.49 | 82.01 | 98.49 | 88.53 | 9 |
|  | ILI rate | 96.23 | 94.29 | 81.63 | 98.95 | 90.51 | 5 |
|  | Influenza incidence | 99.21 | 95.15 | 82.68 | 99.81 | 94.36 | 5 |
| **South-East Asia** | IV Positive rate | 56.90 | 63.30 | 27.03 | 86.01 | 20.20 | 9 |
| **Temperate South America** | IV Positive rate | 83.55 | 83.97 | 63.06 | 93.97 | 67.52 | 9 |
|  | ILI rate | 75.71 | 63.22 | 39.91 | 88.97 | 38.92 | 8 |
|  | Influenza incidence | 88.26 | 87.43 | 69.10 | 95.90 | 75.69 | 8 |
| **Tropical South America** | IV Positive rate | 71.07 | 66.98 | 36.73 | 89.57 | 38.05 | 8 |
| **Western Africa** | IV Positive rate | 58.66 | 88.18 | 50.72 | 91.13 | 46.84 | 9 |
| **Western Asia** | IV Positive rate | 76.07 | 79.49 | 53.19 | 91.55 | 55.56 | 9 |

**Supplementary Figure legend**

Supplementary Figure 1. Classification of the 18 WHO global influenza transmission zones.

Supplementary Figure 2. Geographic distribution of influenza surveillance indicators across global transmission zones.

*Note: (A) Mean IV positivity rate; (B) Mean ILI rate; (C) Mean influenza incidence; (D) Total number of influenza-positive cases; (E) Total number of ILI cases.*
